# Design of protein-binding peptides with controlled binding affinity: the case of SARS-CoV-2 receptor binding domain and angiotensin-converting enzyme 2 derived peptides

**DOI:** 10.3389/fmolb.2023.1332359

**Published:** 2024-01-05

**Authors:** Giacomo Parisi, Roberta Piacentini, Alessio Incocciati, Alessandra Bonamore, Alberto Macone, Jakob Rupert, Elsa Zacco, Mattia Miotto, Edoardo Milanetti, Gian Gaetano Tartaglia, Giancarlo Ruocco, Alberto Boffi, Lorenzo Di Rienzo

**Affiliations:** ^1^ Department of Basic and Applied Sciences for Engineering (SBAI), Università“Sapienza”, Roma, Italy; ^2^ Department of Biochemical Sciences “Alessandro Rossi Fanelli”, Università“Sapienza”, Roma, Italy; ^3^ Department of Biology and Biotechnologies “Charles Darwin”, Università“Sapienza”, Roma, Italy; ^4^ Centre for Human Technologies (CHT), Istituto Italiano di Tecnologia (IIT), Genova, Italy; ^5^ Center for Life Nano and Neuro Science, Istituto Italiano di Tecnologia (IIT), Roma, Italy; ^6^ Department of Physics, Università“Sapienza”, Roma, Italy

**Keywords:** peptide design, molecular dynamics simulation, biolayer interferometry (BLI), shape complementarity, ferritin-based nanoparticle

## Abstract

The development of methods able to modulate the binding affinity between proteins and peptides is of paramount biotechnological interest in view of a vast range of applications that imply designed polypeptides capable to impair or favour Protein-Protein Interactions. Here, we applied a peptide design algorithm based on shape complementarity optimization and electrostatic compatibility and provided the first experimental *in vitro* proof of the efficacy of the design algorithm. Focusing on the interaction between the SARS-CoV-2 Spike Receptor-Binding Domain (RBD) and the human angiotensin-converting enzyme 2 (ACE2) receptor, we extracted a 23-residues long peptide that structurally mimics the major interacting portion of the ACE2 receptor and designed *in silico* five mutants of such a peptide with a modulated affinity. Remarkably, experimental K_D_ measurements, conducted using biolayer interferometry, matched the *in silico* predictions. Moreover, we investigated the molecular determinants that govern the variation in binding affinity through molecular dynamics simulation, by identifying the mechanisms driving the different values of binding affinity at a single residue level. Finally, the peptide sequence with the highest affinity, in comparison with the wild type peptide, was expressed as a fusion protein with human H ferritin (HFt) 24-mer. Solution measurements performed on the latter constructs confirmed that peptides still exhibited the expected trend, thereby enhancing their efficacy in RBD binding. Altogether, these results indicate the high potentiality of this general method in developing potent high-affinity vectors for hindering/enhancing protein-protein associations.

## 1 Introduction

Protein-protein interactions (PPIs) play a pivotal role in numerous biological processes. Within these interactions, selected mutations at the interfaces can lead to significant changes in binding affinity, resulting in physiological or pathological phenotypes (Forbes et al., 2015; Landrum et al., 2016). Indeed, the stability of PPIs is the result of a complex fine-tuning of chemical-physical properties at the interfaces and entropic effects (Vangone and Bonvin, 2015; Desantis et al., 2022), making the substitution of even just one residue potentially disruptive. Therefore, in the last years, several computational methods have been developed to predict the effects of mutations on binding, based on a wide variety of techniques (Moretti et al., 2013; Brender and Zhang, 2015; Geng et al., 2019; Rodrigues et al., 2019). In addition, it has been recently presented a protocol for amino acid refinement through a computational method for the design of peptides and proteins interface (Ochoa et al., 2021).

A paramount example of such a PPI is the complex formation between the SARS-CoV-2 RBD and the human ACE2 receptor protein. This interaction is crucial for viral infection, as it triggers a cascade of events ultimately leading to viral entry into the host cell (Walls et al., 2020). Also in this case, mutations in the RBD or ACE2 interface have been shown to affect virus infectivity and disease conditions (Barton et al., 2021). Considering the significance of this interaction, considerable efforts have been devoted to the development of computational methods for predicting mutation effects in the RBD-ACE2 interface (Zou et al., 2020; Bai et al., 2021; Miotto et al., 2022; Miotto et al., 2023).

To date, several experimental structures of the complex between ACE2 and the receptor-binding domain of the SARS-CoV-2 Spike protein have been determined, providing the structural basis for the specific interaction mechanism and highlighting the critical residues involved in the complex formation (Shang et al., 2020). Interestingly, the peptide “IEEQAKTFLDKFNHEAEDLFYQSSLASWNYNTN” (residues 21–53) appears to mimic the major interacting portion of the ACE2 receptor to the SARS-CoV-2 Spike RBD (Kuznetsov et al., 2022). Therefore, it is not surprising that peptides of different lengths, including residues 24 to 53 of the ACE2 receptor, have commonly been reported to exhibit high affinity binding to various regions of the SARS-CoV-2 Spike protein (de Campos et al., 2021; Larue et al., 2021). Many of these peptides, derived from the N-terminal α-helix of ACE2, have been tested in response to the SARS-CoV-2 pandemic (Tzotzos, 2022). Since the SARS-CoV-2 virus enters cells through the interaction between the Spike glycoprotein and ACE2 ectodomain, disrupting the Spike/ACE2 interaction represents a major target for preventing cell infection (Gheblawi et al., 2020; Papageorgiou and Mohsin, 2020).

In the present investigation, our focus was devoted to the exploration of the α-helix peptide derived from the N-terminus of the ACE2 sequence, specifically encompassing residues 21–43, in its interactions with the RBD protein. Upon application of a protocol design discussed previously (Di Rienzo et al., 2021a), we were able to generate a set of peptide mutants with a modulated affinity with Spike protein. Such a protocol is based, beyond a coarse-grained evaluation of electrostatics compatibility, on the application of the 2D Zernike formalism, to obtain a compact representation of the local shape of molecular surfaces (Milanetti et al., 2021). In this framework, the geometry of a molecular region is described by an ordered set of numbers: this ensures an easy evaluation of the shape complementarity between two molecular regions by calculating the euclidean distance between the corresponding Zernike descriptors. Therefore, when a residue is substituted, it is possible to evaluate whether the shape of the mutated peptide is more complementary with the molecular partner. In the past years, this formalism has proven its efficacy in similar optimization protocols (Di Rienzo et al., 2020; De Lauro et al., 2022; Di Rienzo et al., 2023a), or more in general to evaluate the local similarity or complementarity (Venkatraman et al., 2009; Daberdaku and Ferrari, 2018; 2019; Miotto et al., 2021; Di Rienzo et al., 2022; Piacentini et al., 2022; Di Rienzo et al., 2023b). Hence, we generated a set of five ACE2-derived peptide mutants, four of them endowed with predicted higher affinity for the RBD protein with respect to the wildtype (WT) peptide and one with predicted lower affinity.

Initially, we confirmed, with extensive molecular dynamics simulations of the six peptides (WT + five mutants), that even when the peptides are extracted from the whole ACE2 structure, they maintain the elongated α-helix structure. The peptides were subsequently synthesized and subjected to rigorous *in vitro* testing using biolayer interferometry methods (BLI). These analyses enabled us to experimentally determine the binding affinity between the peptides and the RBD protein, strongly confirming the accuracy of our computational predictions. Thus, to gain insight into the molecular mechanisms driving the increase of the binding affinity we performed molecular dynamics simulations of the complexes between RBD and each of the six peptides.

Finally, as a proof of concept, we genetically fused either the wild-type ACE2 peptide or the optimized peptide with the highest affinity for RBD at the N-terminus of human H ferritin 24-mer. Ferritin is a naturally occurring human protein that thanks to its endogenous nature ensures excellent biocompatibility, biodegradability, and low toxicity, essential features for clinical applications. Its symmetrical spherical architecture, high thermal stability, self-assembly ability, and ease of production in recombinant form make it a promising platform for drug delivery and vaccine development. These structural features make it an ideal candidate for clinical nanocarrier applications. The fusion with ferritin aims to overcome the limitations associated with free peptides, such as rapid renal clearance and reduced bioavailability, by leveraging the multivalent effect of the ferritin’s 24-meric structure. This strategy has the potential to significantly enhance the therapeutic efficacy of peptide-based treatments against SARS-CoV-2 by improving their stability, bioavailability, and virus neutralization capacity. Prior research on ferritin-based drug delivery systems (N. Song, et al., 2021; Palombarini et al., 2020), and in the context of SARS-CoV-2 treatment and vaccine development (Kalathiya et al., 2021; Khoshnejad et al., 2018; Kim et al., 2017; Kim et al., 2022; Kanekiyo et al., 2013; Powell et al., 2021; Sliepen et al., 2015), provides a foundation for the investigation reported in the present paper.

## 2 Results and discussion

### 2.1 Computational analysis of the ACE2-derived peptides

**FIGURE 1:**
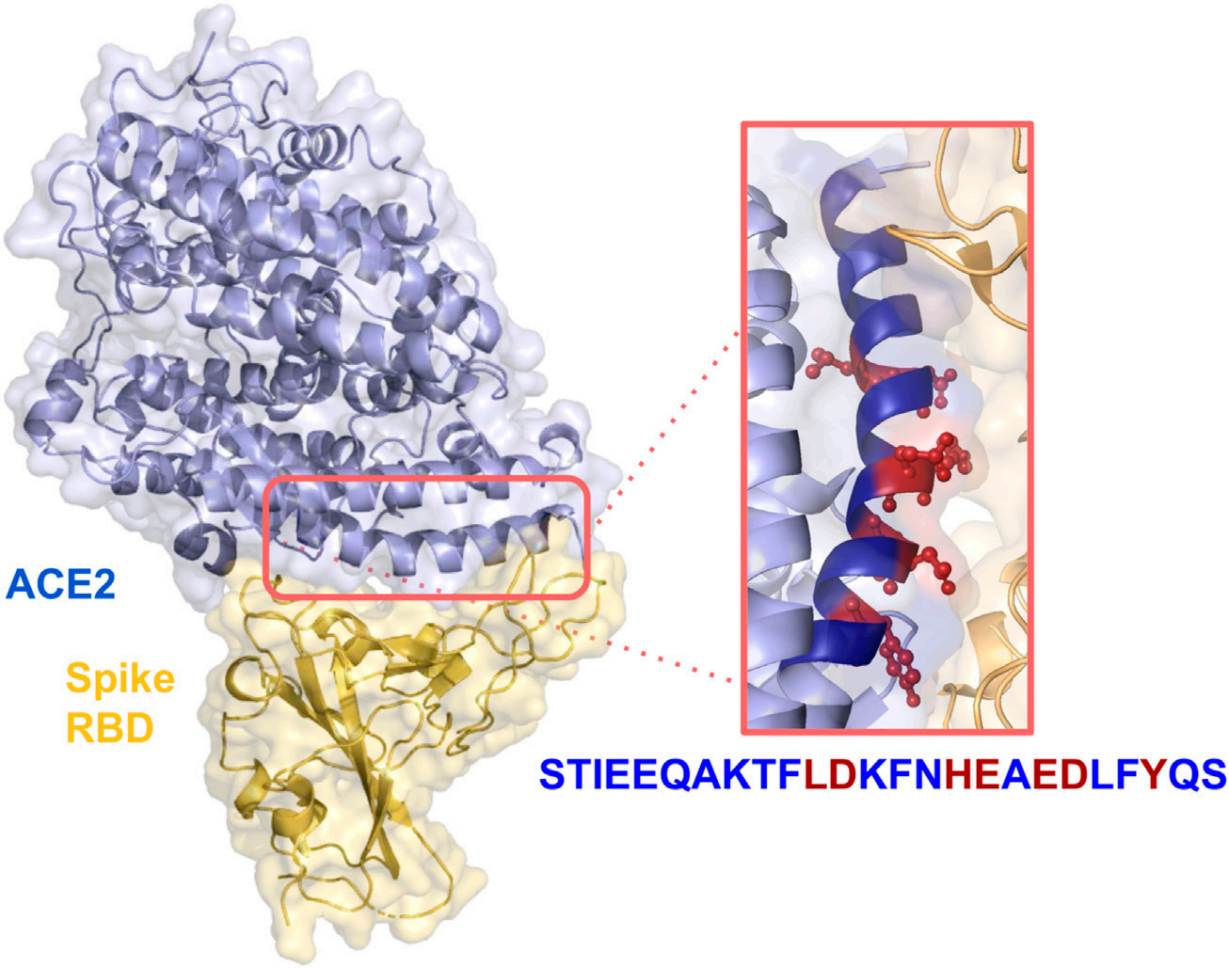
Crystal structure of SARS-CoV-2 spike receptor-binding domain bound with ACE2 (PDB id: 6VW1). Relevant side chains of the residues in the α-helix are shown in the orange box.

In a previous paper, we presented a designed computational protocol aiming to generate soluble-ACE2 mutants with increased or decreased affinity with SARS-CoV-2 Spike (Di Rienzo et al., 2021b). Here, we applied the same protocol, restraining the application onto residues into the α-helix peptide range 21–43. The spatial arrangement of the peptide within the ACE2-RBD complex can be observed in Figure 1.

This protocol performs the substitution in each position of the WT molecule with the 19 other possible lateral chains, selecting, among the substitutions with compatible electrostatics, the ones driving the highest increase in shape complementarity. Using this approach, we can generate peptides with controlled compatibility with the partner and with a defined number of mutations with respect to the wild type. We decided to set the number of mutations to three to preserve the global features of the peptide. Hence, we selected the four mutated peptide sequences (HA1, HA2, HA3, and HA4) that exhibited the highest nominal affinities towards the RBD compared to the WT sequence. Moreover, inverting the function to optimize in the computational protocol, we selected the peptide sequence with the predicted lowest affinity (LA1) to work as a control. The sequences of each of the six peptides are detailed in Table 1, where the specific mutated amino acids are highlighted in red.

**TABLE 1:** Sequences of the wild type α-helix peptide and the list of mutated sequences HA1-4 and LA1. The mutated amino acids are highlighted in red.

WT	STIEEQAKTFLDKFNHEAEDLFYQS
HA1	STIEEQAKTFLDKFNILALDLFYQS
HA2	STIEEQAKTFLDKFNVLALDLFYQS
HA3	STIEEQAKTFDDKFNILAEDLFYQS
HA4	STIEEQAKTFYDKFNVLAEDLFYQS
LA1	STIEEQAKTFLGKFNHEAEYLFRQS

**FIGURE 2:**
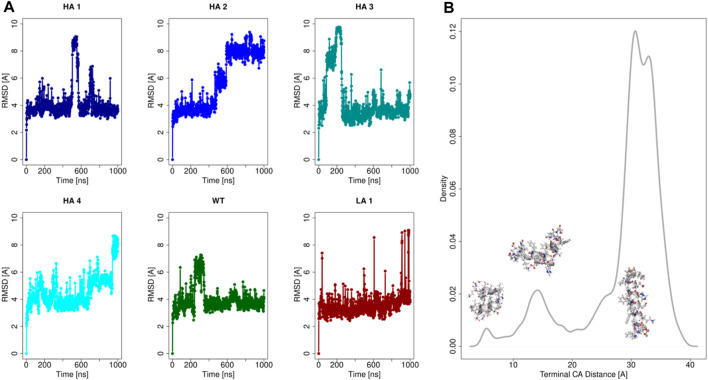
Analysis of the peptides molecular dynamics. **(A)** Root Mean Square Deviation (RMSD) as a function of time with respect to the initial configuration for each of the six peptides. **(B)** Density distribution of the spatial distance between the terminal α-carbon, for all of six peptides simulations.

To characterize the properties of these six peptide sequences, we performed molecular dynamics simulations. In particular, each of the 6 peptides has been extensively simulated in water as a monomer (1μs-long each). Indeed, it has to be noted that we performed the shape complementarity optimization using the elongated α-helix form of the peptide when inserted in the ACE2 structure. Therefore, the peptides’ behavior in water was assessed to ensure the applicability of our computational optimization protocol. Relevant results are summarized in Figure 2.

In Panel (a) we reported the Root Mean Square Deviation (RMSD) of the simulations of all peptides. It emerges that all the mutants at the equilibrium can assume mainly three conformations in dynamics at room temperature, characterized by three equilibrium RMSD values. These three conformational states are characterized by three different levels of structure compactness, as demonstrated by the distribution reported in panel (b). Indeed, for each frame of all the molecular dynamics simulations, the distance between the peptides terminal C-alpha was calculated and its distribution is shown in Figure 2.

The simulations commence from the extended conformation, and the low RMSD values, approximately 4 Å, indicate frames where the peptide structure remains extended. Occasionally, albeit for brief periods, peptides may self-fold, reducing the distance between their terminal residues (refer to the distribution in Figure 2B). Consequently, high RMSD values, computed concerning the initial configuration of molecular dynamics, are observed in frames where the peptide undergoes further compaction. Three peaks are present in correspondence to the three main accessible configurations for the peptides. More importantly, the stretched configuration (the one with the highest value of terminal distance) is much more frequent, testifying its advantage in terms of free energy.

This result demonstrated that at equilibrium all peptides spend a significant amount of time in a stretched form, as previously observed (Baig et al., 2020). Therefore, the optimization performed using the elongated form of the peptide has in the ACE2 structure is meaningful and well-defined.

### 2.2 Spike-peptide complexes: experimental binding kinetics measurements and molecular dynamics simulation

Binding kinetics measurements were carried out by using the biolayer interferometry technique, in order to assess the affinity between the ACE2-derived peptides and RBD fragment in solution. This approach has been extensively employed in the investigation of antibody-antigen interactions (Concepcion et al., 2009; Kamat and Rafique, 2017; Petersen, 2017), with a specific emphasis on the interactions between SARS-CoV-2 variants and antibodies (Dzimianski et al., 2020; Ginex et al., 2022; Wang et al., 2022; Wang et al., 2023).

**FIGURE 3:**
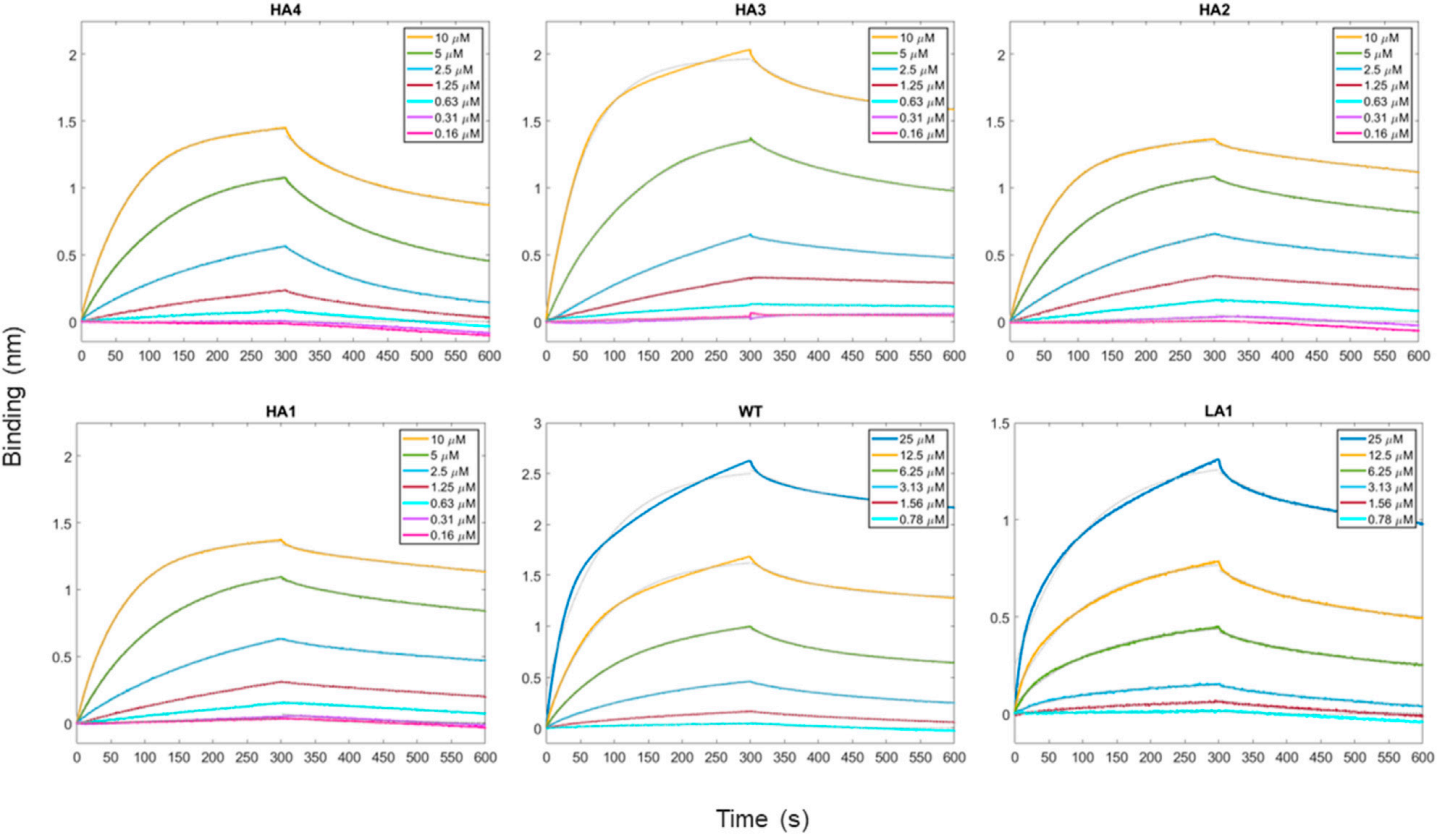
Time-course of the reaction between peptides and RBD as the analyte protein in solution at various concentrations. For all measurements, a 300 s time period was set for both association and dissociation steps. Peptides have been loaded first at fixed concentration (50 μg/mL) on biosensors initialized with streptavidin molecule.

In the present study, we performed biotinylation of the WT, HA4, HA3, HA2, HA1, and LA1 peptides to enable their immobilization on the biosensor surface. Subsequently, we conducted kinetic measurements (as depicted in Figure 3) at various concentrations of RBD ranging from 100 μM to 0.16 µM. In Table 2 we presented the obtained K_D_ values, representing the affinity between the peptides and RBD, as well as the shape complementarity balance predicted by the computational protocol.

**TABLE 2:** The values of K_D_ with standard errors from data analysis of BLI assay traces are reported in the first row. K_D_ were obtained directly within the Octet software. In the second row we report the complementarity balance, that is the increase in shape complementarity, in terms of distance between Zernike descriptors, with respect to the Wild Type.

Peptide	LA1	WT	HA4	HA3	HA2	HA1
K_D_ (µM)	74.0 ± 7.2	33.0 ± 1.9	7.2 ± 1.0	5.0 ± 1.0	2.70 ± 0.45	2.20 ± 0.44
Complementarity Balance	−1.092	0.0	0.275	0.276	0.310	0.329

The shape complementarity balance describes the increase in shape complementarity due to the residue substitutions with respect to the wild type, as calculated with the euclidean distance between the Zernike descriptors of the peptides and the Spike binding site. Notably, the affinity constants are in the micromolar range and exhibit the same trend as the one predicted via the computational approach.

Specifically, the HA4, HA3, HA2, and HA1 mutants displayed increased affinity towards RBD compared to the WT peptide. Conversely, LA1 exhibited significantly lower affinity.

**FIGURE 4:**
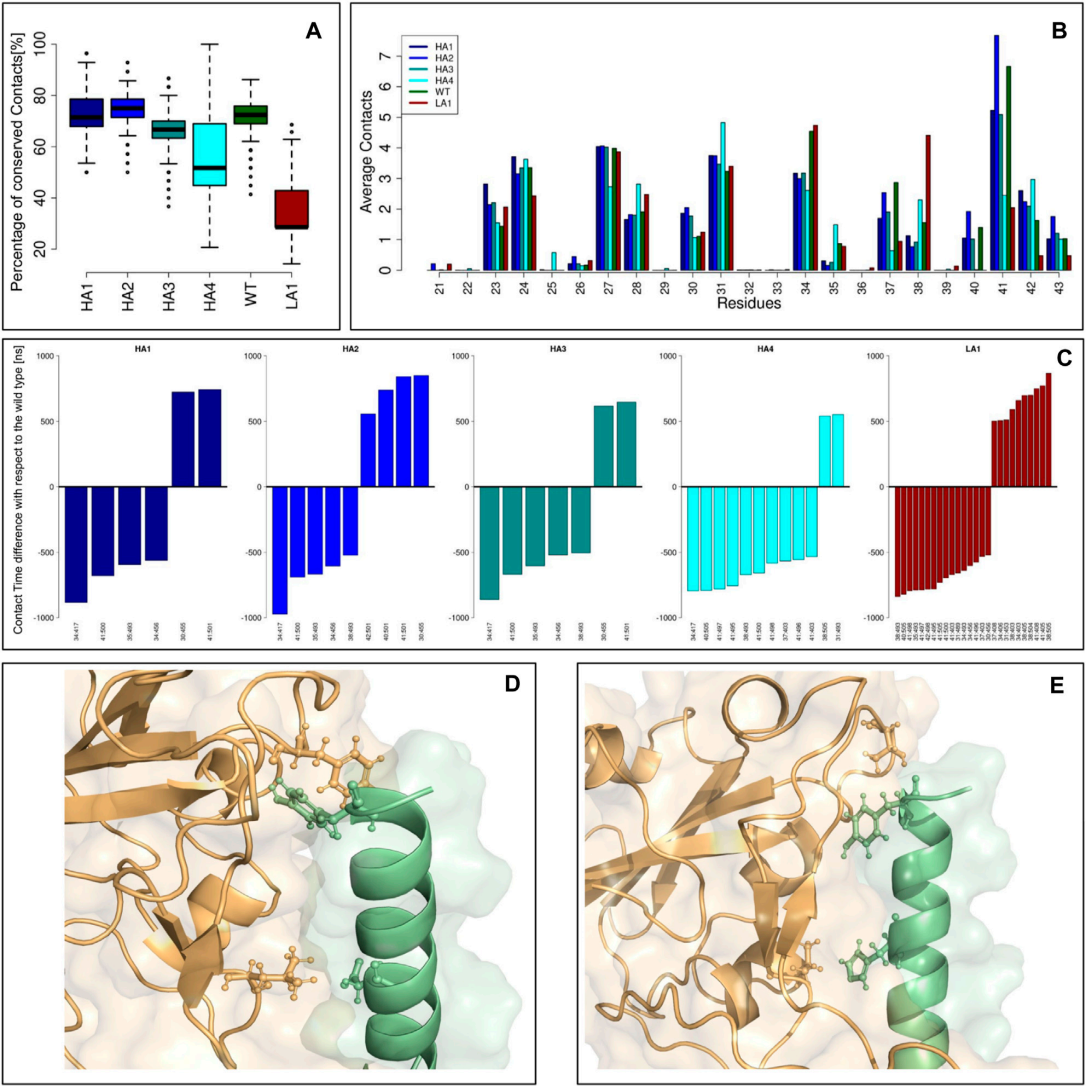
Peptide-spike complexes molecular dynamics. **(A)** Boxplots describing the percentage of conserved contacts at the equilibrium, with respect to those observed in the starting structure. **(B)** The average contacts each peptide residue made during the equilibrium simulation frames. Each color represents a specific mutant peptide-Spike complex. **(C)** Intermolecular links characterized by an occurrence difference higher than 50% with respect to the wild type. **(D)** Molecular representation of the Peptide-Spike complex with highlighted the 30:455 and 41:501 links, acquired by the high affinity mutants. **(E)** Molecular representation of the Peptide-Spike complex with highlighted the 34:417 and 41:500 links, lost by the high affinity mutants.

In order to further understand the molecular mechanisms driving the peptide recognition process, we performed six 1μs-long molecular dynamics simulations of the SARS-CoV-2 Spike protein in complex with each of the peptides. The complexes were built by extracting the Spike-peptide complex from the experimental Spike-ACE2 complex and substituting the appropriate mutated residues within the peptide. The main results of the simulations are summarized in Figure 4 and can be discussed as follows.

Defining a residue-residue intermolecular contact as a pair of Spike-Peptide residues with at least one of their atom-atom pairs closer than 4 A, we first studied the intermolecular contacts conserved along the dynamics, as an indicator of the stability of the molecular complex. In particular, in panel (a) we reported the boxplots describing the percentage of conserved contacts at the equilibrium, with respect to those observed in the starting structure. Notably, the low-affinity mutant displays a low number of conserved contacts as a consequence of its reduced complex stability, in full agreement with the results of the biochemical essays. Another proof of this behavior can be searched on the RMSD trend over time for each of the 6 peptides simulated in complex with the Spike protein (Supplementary Figure S1). All peptides with increased affinity and the wild-type peptide exhibit significant stability, evidenced by the consistent RMSD trend. Conversely, in the simulation of the peptide with reduced affinity, we observe a moment when the peptide undergoes a conformational change (RMSD peak) before readjusting to a new position. At that moment, the peptide shifts, losing many of its initial intermolecular contacts (as depicted in Figure 4A), confirming that the design has rendered it unsuitable for this situation.

We thus moved to the analysis of the peptide residues responsible for the interaction with Spike. The average number of intermolecular contacts of each residue, executed during the molecular dynamics, were calculated and shown in panel (b). Interestingly, some residues exhibited a different behavior depending on which mutant we were considering. A very high number of contacts are undertaken by residue 41 in both WT and HA mutants, whereas the LA mutant dramatically reduces the reactivity of such a residue. Conversely, residue 34 is characterized by a high number of contacts in WT and LA peptides, whereas in all the HA mutants this residue lessens the interaction with Spike.

Finally, in panel (c), we studied which intermolecular links differ the most between the WT and the mutant peptides. Indeed, for each residue-residue intermolecular pair, we calculated the occurrence in both WT and mutant simulation frames: we reported for each mutant peptide the barplots indicating the residue-residue links that highlight a difference with the Wild Type higher than 50%. In other words, each bar represents a contact with Spike that has been acquired or lost by a mutant with respect to the ones characterizing the Wild Type: these contacts can be responsible for the variation in binding affinity these mutants exhibit. We noted that some common features are present in the increased affinity mutants simulations. On one hand, the HA mutants acquired, with respect to the WT complex, the interactions 30:455 and 41:501 (represented in panel (d)), whereas they lost the interactions 34:417 and 41:500 (panel (e)). It has to be noted that among all the residues involved in these dramatic changes, only residue 34 undergoes a mutation (H34I or H34V): this result proves the capability of the design protocol to compactly summarize the overall interface features.

### 2.3 ACE2-derived peptides/ferritin chimeric nanoparticles

Based on the findings obtained from the individual peptides, we sought to demonstrate the feasibility of fusing these peptides with human ferritin H as a proof of concept. Indeed, the application of the peptides alone for future therapeutic purposes is limited due to challenges related to poor bioavailability and high clearance rates. To overcome these limitations, one strategy involves combining the peptides with nanoparticles of diverse compositions. Nanoparticles (NPs) have demonstrated their potential as conjugate scaffolds, enhancing peptide functionality and leveraging their intrinsic properties, often leading to synergistic effects (Jeong et al., 2018).

Protein-based nanoparticles are particularly suitable, given their biocompatibility, ease of producing monodisperse forms through recombinant techniques, and the ability to be modulated via genetic engineering approaches. Among these protein nanoparticles, ferritin serves as an ideal platform for diagnostic and therapeutic applications (Calisti et al., 2018; Palombarini et al., 2021; Affatigato et al., 2023). Comprised of 24 subunits, human ferritin forms a nanosphere with a diameter of 12 nm. Modifying a single subunit enables the functionalization of the entire nanoparticle (Incocciati et al., 2023).

In the case of ACE2-derived peptides, fusing the peptide sequence to the N-terminus of human H ferritin offers the advantage of generating a chimeric protein that presents 24 peptides on its surface. This arrangement leads to a multivalent effect while concurrently reducing peptide clearance.

In our specific case, we opted to genetically fuse both the WT sequence and the HA1 sequence, which exhibits the highest affinity for RBD, to the N-terminus of human H ferritin. To ensure structural integrity, a flexible spacer consisting of four glycine residues was included in the fusion construct. WT-HFt and HA1-HFt constructs were overexpressed in *Escherichia coli*. Interestingly, the WT-HFt was successfully expressed in a soluble form, while, HA1-HFt was obtained in inclusion bodies, necessitating additional steps for purification. Nevertheless, both proteins were purified to a high degree of purity (Supplementary Figure S3), enabling subsequent structural and functional characterization.

**FIGURE 5:**
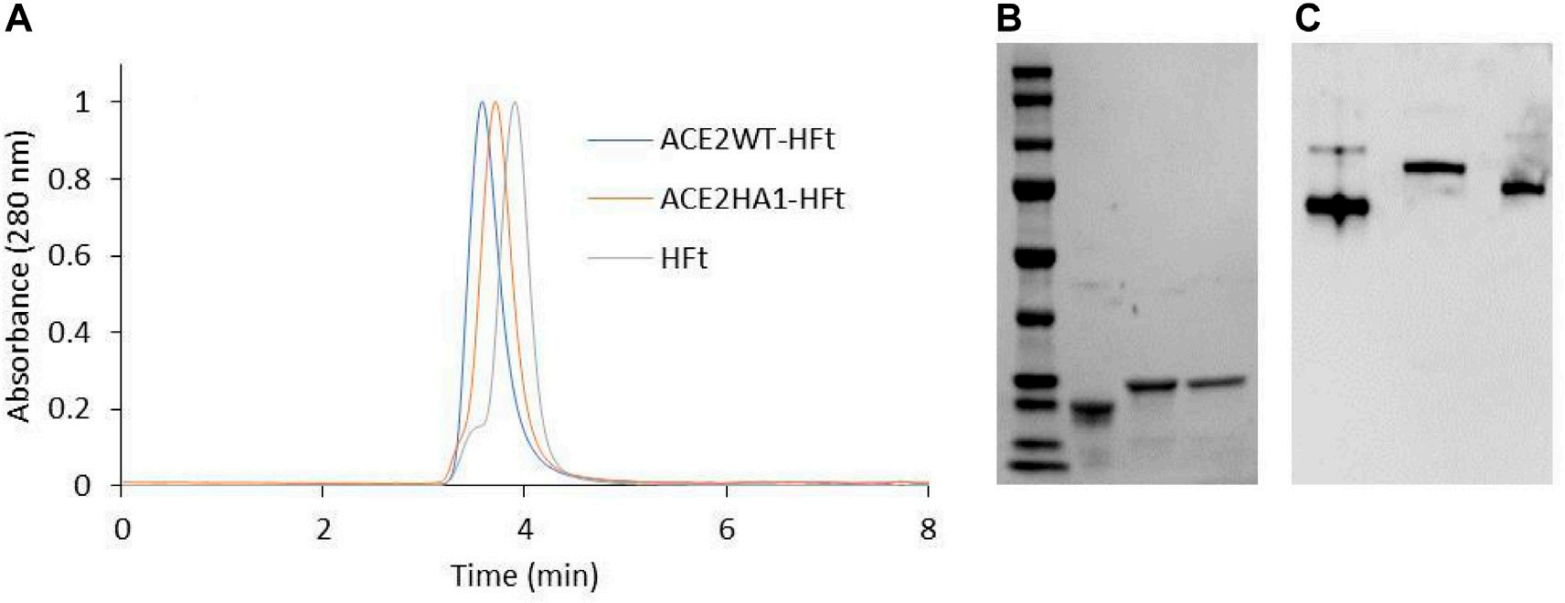
Biochemical characterization of ACE2WT-HFt and ACE2HA1-HFt compared to HFt. **(A)** HP-SEC analysis. **(B)** SDS-PAGE (lane 1: marker, lane 2: HFt, lane3: ACE2WT-HFt, lane 4: ACE2HA1-HFt). **(C)** native gel electrophoresis (lane1: HFt, lane2: ACE2WT-HFt, lane 3: ACE2HA1-HFt).

SDS-PAGE analysis demonstrates that the individual subunits of each chimeric ferritin exhibit the expected increase in molecular weight due to the addition of the peptide. Moreover, *High-performance size exclusion chromatography* (HP-SEC) analysis and native gel electrophoresis confirm the correct assembly of these ferritins into their 24mer form (Incocciati et al., 2022). The HPLC elution profile reveals a higher molecular weight for the chimeric ferritins compared to human H ferritin. As shown in Figure 5A, the WT-HFt variant displays a weight increase of 77,266 Da (retention time 3.53 min) while the HA1-HFt shows an increase of 75,924 Da (retention time 3.67 min).

**FIGURE 6:**
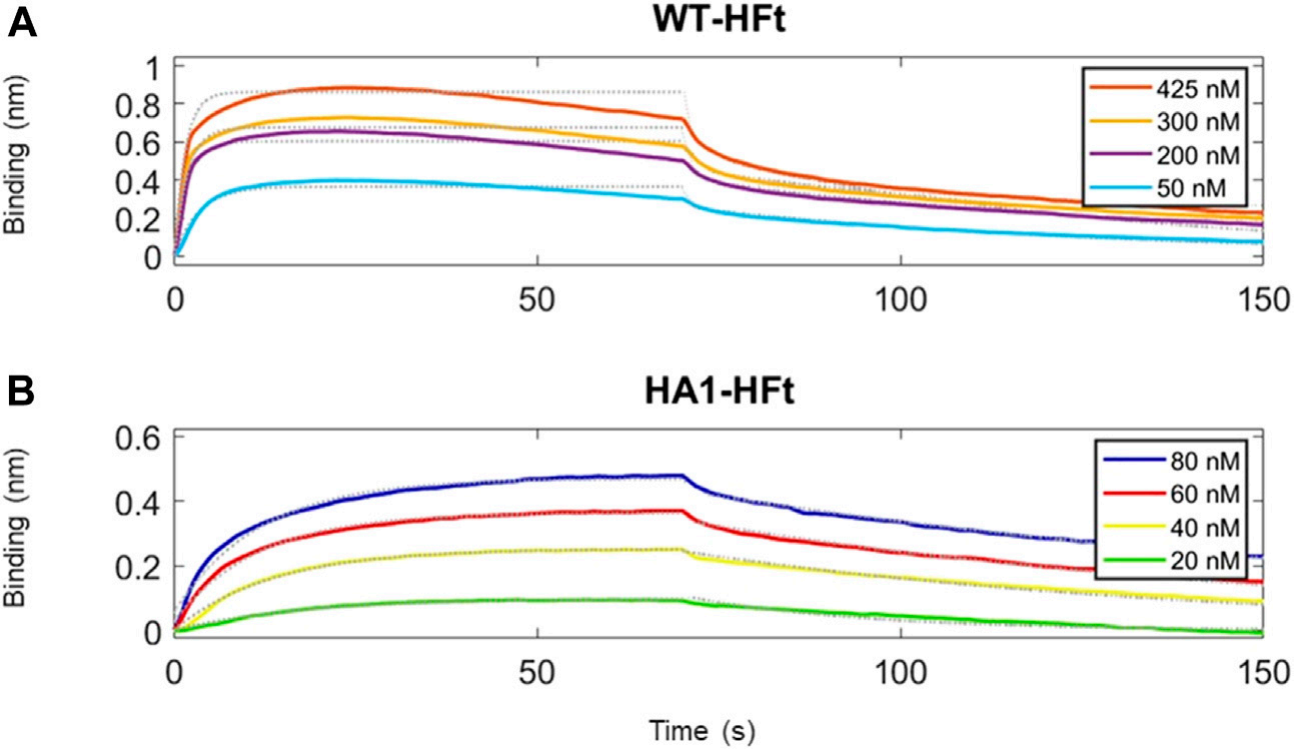
Time courses of the reaction between RBD and ferritin constructs. RBD protein has been loaded first at fixed concentration (50 μg/mL) on Ni-NTA biosensors. **(A)** ferritin with WT peptide construct is the analyte protein in solution at various concentrations. We set a 70 s time interval for the association step and 80 s for dissociation. **(B)** ferritin with HA1 peptide construct is the analyte protein in solution at various concentrations. Association and dissociation steps have duration respectively of 70 s and 80 s. Temperature was 25 °C.

Similarly, the native gel electrophoresis demonstrates that the chimeric variants migrate slower than ferritin alone (Figures 5B, C), as expected. The disparities in electrophoretic mobility between the two mutants can be attributed to their differing masses and charges. After characterization, the binding capacity of the two chimeric ferritins to the Spike RBD was assessed. The analysis was performed using the BLI technique, wherein His-tagged RBD was immobilized on the Ni-NTA biosensor, and the concentrations of the chimeric ferritins were systematically varied. Subsequently, the binding kinetics were examined, and the results are illustrated in Figure 6. The analysis of the kinetic plots revealed a dissociation constant (KD) of 10.81 nM for WT-HFt and 8.32 nM for HA1-HFt, providing quantitative insights into the affinity of the respective constructs for RBD. A summary of these results can be find in Table 3.

**TABLE 3:** Values of K_D_ with standard errors from data analysis of BLI assay traces.

Complex	WT-HFt	HA1-HFt
K_D_ (nM)	10.81 ± 0.06	8.32 ± 0.03

## 3 Conclusion

The key output of the present article is related to the *in silico* testing and *in vitro* experimental validation of a method for the design of polypeptide mutants with optimized binding capabilities to their target protein. Such achievement paves the way for a wide range of possible applications in modulating the binding affinity of selected protein-protein interactions by rational prediction of site-selected mutants using a simple algorithm based on Zernike polynomials descriptors. Computational studies and *in vitro* experiments have been focused on the interaction between the SARS-CoV-2 Spike protein and its main cellular receptor, ACE2 as this interaction stands as a prime and extensively studied example of protein-protein binding and has been instrumental in understanding the mechanisms behind viral cell invasion at molecular level. Leveraging the compatibility of the Spike-ACE2 interface, we explored the use of soluble ACE2 fragments as potential inhibitors of such a paramount example of protein-protein interaction.

In this study, we selected a peptide representing ACE2’s major interaction region and aimed to enhance its affinity with the Spike receptor-binding domain (RDB) for more effective inhibition. Thus far, we used a computational mutagenesis protocol that assessed shape complementarity and electrostatic interactions at the peptide-protein interface.

In other preceding works, we have demonstrated the significant role that shapes complementarity and electrostatic compatibility play at the interface, highlighting how the Zernike formalism is refined enough to capture this aspect (Di Rienzo et al., 2021a; De Lauro et al., 2022; Desantis et al., 2022; Grassmann et al., 2023). Undoubtedly, other physicochemical features are crucial for a comprehensive characterization of the compatibility between two protein interfaces. In our plans, we intend to incorporate these aspects into the algorithm cost function to account for them. However, these two characteristics successfully describe at least a substantial portion of this phenomenon, as evidenced by the success of our design protocol. In fact, not only are we able to achieve peptides with improved binding affinity, but we also obtain a negative control with worsened binding affinity due to compromised electrostatic compatibility and deteriorated shape complementarity. Indeed, our results identified peptide mutants with predicted changes in binding affinity as compared to the wild type, and these findings were confirmed in a set of *in vitro* experiments. Most significantly, the observed results mark the first successful *in vitro* experimental validation of our protein design algorithm.

In order to complete the picture, wild-type and optimized peptides were fused to the N-terminus of human H ferritin 24-mer, a most popular protein carrier that was recently demonstrated to be a preferred multivalent epitope displaying agent in the construction of protein-based nanoparticles for efficient vaccine constructs assembly. Further *in vivo* studies will be necessary in order to assess the reduced clearance of the present construct with respect to isolated peptides upon administration in appropriate experimental animal systems. In this context, the multivalency effect of peptide-fused ferritin 24-mer was also investigated in its binding to immobilized RBD protein using biolayer interferometry measurements. Experimental data demonstrated a 250-fold increase in the binding affinity of the peptide-HFt constructs with respect to the isolated peptides in biolayer interferometry experiments.

In conclusion, the body of experimental results provides the first strong experimental evidence for the effectiveness of the reported computational design protocol. The method thus proved to be highly adaptable and very efficient, thus setting the stage for a comprehensive approach to modulate a wide range of protein-protein interactions.

## 4 Materials and methods

### 4.1 Peptides and chimeric ferritin production

The wild-type RBD of SARS-CoV-2 spike protein was provided by GenScript. The protein is a C-term HIS-tagged recombinant protein with a predicted molecular weight of 30 kD. The storage buffer was phosphate-buffered saline at pH 7.2, and the calculated pI was 8.91; the stock concentration was 0.89 mg/mL according to the manufacturer.

ACE2-derived peptides WT, HA1, HA2, HA3, HA4, LA1 were synthesized by GenScript and provided in the lyophilized form. Before use, they were resuspended in PBS buffer containing either 3% NH_4_OH or 5% NH_3_ (depending on the total net charge of the peptides). The final stock concentration for each peptide was 5 mg/mL.

The synthetic genes encoding for HA1-HFt and WT-HFt chimeric proteins were synthesized by GenScript and optimized for expression in *Escherichia coli* cells. Peptide sequences were inserted at the N-terminal region of the ferritin through a 4 glycine-spacer. The genes were then subcloned into pET22 b vector e transformed in BL21 (DE3) competent cells.

HFt was expressed and purified as previously reported (Palombarini et al., 2019).

*WT-HFt purification protocol*: Bacterial paste from 1 L culture was resuspended in 50 mL of 20 mM sodium phosphate buffer, pH 7.4 containing 150 mM NaCl and protease inhibitors (Roche^©^) and disrupted by sonication. The soluble fraction was precipitated with 20% (NH_4_)_2_SO_4_ for 1 h at 4°C. The pellet was resuspended in 20 mM sodium phosphate buffer pH 7.4 containing 150 mM NaCl and extensively dialyzed versus the same buffer overnight at 4 °C. Finally, the protein was loaded onto a HiLoad 26/600 Superdex 200 pg column equilibrated with 20 mM sodium phosphate buffer pH 7.4 containing 150 mM NaCl, using an AKTA-Pure apparatus (Cytiva).

*HA1-HFt purification protocol*: Bacterial paste from 1 L culture was resuspended in 50 mL of 50 mM Tris/glycine buffer, pH 7.5 containing 150 mM NaCl, 0.5 mM TCEP and protease inhibitors (Roche^©^) and disrupted by sonication. The insoluble fraction was resuspended in 50 mL of 100 mM Tris/glycine buffer pH 8.5, containing 0.5 mM TCEP and 0.5 M urea. The solution was stirred for 30 min at room temperature. The resulting washed pellet was resuspended in 50 mL of 100 mM Tris/glycine buffer pH 8.5, containing 0.5 mM TCEP and 4 M urea, and stirred for 30 min at room temperature. After centrifugation, the soluble fraction was dialyzed overnight versus 50 mM Tris/glycine buffer pH 9 at 4°C. The protein sample was then dialyzed for 4 h versus sodium phosphate buffer pH 7.4, concentrated, and loaded onto a HiLoad 26/600 Superdex 200 pg as described above.

The protein fractions eluting at the retention time of ferritin were pooled, concentrated using AmiconUltra-15 centrifugal filter devices (100 kDa cut-off), sterile filtered, and stored at 4 °C. Protein concentration was calculated by measuring the UV absorption at 280 nm (ε_280_ = 19,000 M^−1^ cm^−1^ and 20,400 M^−1^ cm^−1^ for HFt and the chimeric proteins, respectively). Protein purity was checked by SDS-PAGE and the correct quaternary assembly was evaluated by high-performance size exclusion chromatography (HP-SEC).

*High-performance size exclusion chromatography.* The purity and aggregation state of protein samples were analyzed by HP-SEC. HP-SEC analyses were performed by means of an Agilent Infinity 1260 HPLC apparatus equipped with UV detectors using an Agilent AdvanceBio SEC 300 A 2.7 um 4.6 × 150 mm column. Isocratic analysis was carried out with 20 mM sodium phosphate buffer pH 7.4 as the mobile phase. The flow rate was 0.7 mL/min over an elution window of 10 min. Ferritin elution was followed using UV detection at 220 nm and 280 nm.

### 4.2 Biolayer interferometry

BLI assays were performed using the Octet N2 system (Sartorius). The interaction between ACE2-derived peptides (WT, HA1, HA2, HA3, HA4, LA1), and RBD was conducted by immobilizing the biotinylated peptides on High Precision Streptavidin 2.0 (SAX2) biosensors, with varying concentrations of RBD in the range of 0.16 µM–100 µM. The peptides were either subjected to a biotinylation reaction using the BTAG biotinylation kit (Sigma-Aldrich) or were provided pre-biotinylated by the manufacturer (GenScript). The interaction between the chimeric proteins WT-HFt (in the range of 425 nM–50 nM) or HA1-HFt (in the range of 80 nM–20 nM) and RBD was performed by immobilizing the His-tagged RBD on nickel nitriloacetic acid (Ni-NTA) biosensors.

The biosensors were first equilibrated for 10 min in 1X kinetic buffer (Sartorius), which consisted of PBS with 0.02% Tween20, 0.1% BSA, and 0.05% NaN_3_. Subsequently, depending on the assay, either ACE2-derived peptide or RBD was loaded onto the corresponding biosensor at a concentration of 50 μg/mL for an appropriate time interval, as indicated in the Sartorius biosensors’ datasheets. The duration of each experimental step was optimized in order to achieve maximum binding capacity in each experiment. When available, the concentration range for each associating protein was chosen based on K_D_ values obtained from the literature or experimentally determined for different scenarios where K_D_ values were unknown.

The recorded data were analyzed using the Octet software to extrapolate the kinetic parameters. All association and dissociation curves were fitted using a single exponential function. Pseudo-first order (PFO) conditions were met when the initial concentration of one of the two reagents was significantly higher (between 50- and 100-fold) than the other (Malatesta, 2005; Kumaraswamy and Tobias, 2015; Müller-Esparza et al., 2020).

### 4.3 Protein surface construction

As a starting point, we used the experimental RBD-Spike complex structure labeled with the PDB code 6vw1 (Shang et al., 2020): it was solved with x-ray crystallography with a resolution of 2.68 A. The structure report ACE2 residues from Serine 19 to Alanine 614 and RBD residues from Asparagine 334 to Proline 527. Starting from the wild type ACE2 sequence, we modeled all the possible point mutations in range 21–43 using Scwrl4 (Krivov et al., 2009). Atomic charges and radii were assigned using PDB2PQR (Dolinsky et al., 2004). Solvent Accessible Surface and are computed using dms software (Richards, 1977).

### 4.4 Computational protocol for peptide design

We applied here the protocol design we developed in a previous publication (Di Rienzo et al., 2021b), restraining the set of mutable residues to the range 21–43.

The main step of the protocol are:

- Starting from the experimental structure, we extracted the surface generated by residue 21–43 from the whole surface and we calculated its 2D Zernike descriptors (20th order, 121 invariant descriptors).
- We calculated the 2D Zernike descriptors regarding the Spike binding site, defined as the set of Spike residues closer than 5 A to any ACE2 atoms.
- We measured the shape complementarity as the Euclidean distance between these two sets of descriptors.
- To assess Coulomb contributions, we developed a Coarse Grained atomic representation. We assigned atomic partial charges and we represented each residue with a bead summarizing the main chain and a bead summarizing the side chain. For each residue they are located on the geometrical center and are charged with the sum of the partial charges of the corresponding atoms. The electrostatic compatibility is computed with a standard Coulomb potential.

The design algorithm works as follows:

- From the wild type ACE2 sequence, we modeled all the possible point mutations in residue range 21–43.
- We accepted only substitutions with non-unfavourable electrostatics modifications. In other words, we accepted only mutations that do not cause an increase in electrostatic energy at the interface.
- We identified among them the best two mutations in terms of shape complementarity increase, as calculated in terms of Zernike descriptors.
- Settled these substitutions, we repeated the computational mutagenesis on all the remaining binding site residues, therefore defining four double site mutants.
- Repeating three times this procedure, we identified eight possible ACE2 mutants characterized by increased shape complementarity and compatible electrostatics.

Analogously, we perform the same procedure aiming in decreasing the shape complementarity. We thus obtained eight mutants with decreased shape complementarity. Finally we selected the best four mutants and the worst mutant in terms of increase and decrease of complementarity, respectively, to be tested experimentally.

### 4.5 Molecular dynamics simulation

We used Gromacs 2021 to run all the molecular dynamics simulations (Abraham et al., 2015). Topologies of the system were built using the CHARMM-27 force field (Brooks et al., 2009). Each molecular system was firstly minimized with the steepest descent algorithm. Thus, we performed the thermalization of the system running sequentially in a NVT and NPT environments 2 0.1ns-long simulations at 2fs time-step. The temperature is set at 300 K with v-rescale thermostat (Bussi et al., 2007) and the pressure (1 bar) is kept constant using Parrinello-Rahman barostat (Parrinello and Rahman, 1980). A cut-off of 1.2 nm was imposed for the evaluation of short-range non-bonded interactions and the Particle Mesh Ewald method (Cheatham et al., 1995) for the long-range electrostatic interactions.

## Data Availability

The raw data supporting the conclusion of this article will be made available by the authors, without undue reservation.
