# Supplementary material for: Design of protein-binding peptides with controlled binding affinity: the case of SARS-CoV-2 receptor binding domain and angiotensin-converting enzyme 2 derived peptides
Source: Front Mol Biosci. 2024 Jan 5;10:1332359. doi: 10.3389/fmolb.2023.1332359 (PMC10797010; doi:10.3389/fmolb.2023.1332359)
Supplement: Supplementary file 1 [file DataSheet1.PDF]

Design of protein-binding peptides with controlled binding affinity: the case of SARS-CoV-2 receptor binding domain and angiotensin-converting enzyme 2 derived peptides

Giacomo Parisi[1,+], Roberta Piacentini[2,+], Alessio Incocciati[2], Alessandra Bonamore[2], Alberto Macone[2], Jakob Rupert[3,4], Elsa Zacco[4], Mattia Miotto[5], Edoardo Milanetti[6,5], Gian Gaetano Tartaglia[3,4], Giancarlo Ruocco[5,6], Alberto Boffi[2], Lorenzo Di Rienzo[5,*].

[+] The authors contributed equally to the present work
* Corresponding author: lorenzo.dirienzo@iit.it

1. Department of basic and applied sciences for engineering (SBAI), Università "Sapienza", Via Antonio Scarpa 16, 00161 Roma, Italy.
2. Department of Biochemical Sciences "Alessandro Rossi Fanelli", Università "Sapienza", P.le Aldo Moro 5, 00185 Roma, Italy.
3. Department of Biology and Biotechnologies "Charles Darwin", Università "Sapienza", P.le Aldo Moro 5, 00185 Roma, Italy.
4. Centre for Human Technologies (CHT), Istituto Italiano di Tecnologia (IIT), 16152 Genova, Italy
5. Center for Life Nano & Neuro Science, Istituto Italiano di Tecnologia (IIT), viale Regina Elena 291, 00161 Roma, Italy.
6. Department of Physics, Università "Sapienza", P.le Aldo Moro 5, 00185 Roma, Italy.

Abstract

The development of methods able to modulate the binding affinity between proteins and peptides is of paramount biotechnological interest in view of a vast range of applications that imply designed polypeptides capable to impair or favour Protein-Protein Interactions. Here, we applied a peptide design algorithm based on shape complementarity optimization and electrostatic compatibility and provided the first experimental in vitro proof of the efficacy of the design algorithm. Focusing on the interaction between the SARS-CoV-2 Spike Receptor-Binding Domain (RBD) and the human angiotensin-converting enzyme 2 (ACE2) receptor, we extracted a 23-residues long peptide that structurally mimics the major interacting portion of the ACE2 receptor and designed in silico five mutants of such a peptide with a modulated affinity. Remarkably, experimental K_D measurements, conducted using biolayer interferometry, matched the in-silico predictions. Moreover, we investigated the molecular determinants that govern the variation in binding affinity through molecular dynamics simulation, by identifying the mechanisms driving the different values of binding affinity at a single residue level. Finally, the peptide sequence with the highest affinity, in comparison with the wild type peptide, was expressed as a fusion protein with human H ferritin (HFt) 24-mer. Solution measurements performed on the latter constructs confirmed that peptides still exhibited the expected trend, thereby enhancing their efficacy in RBD binding. Altogether, these results indicate the high potentiality of this general method in

developing potent high-affinity vectors for hindering/enhancing protein-protein associations.

Supplementary Figures

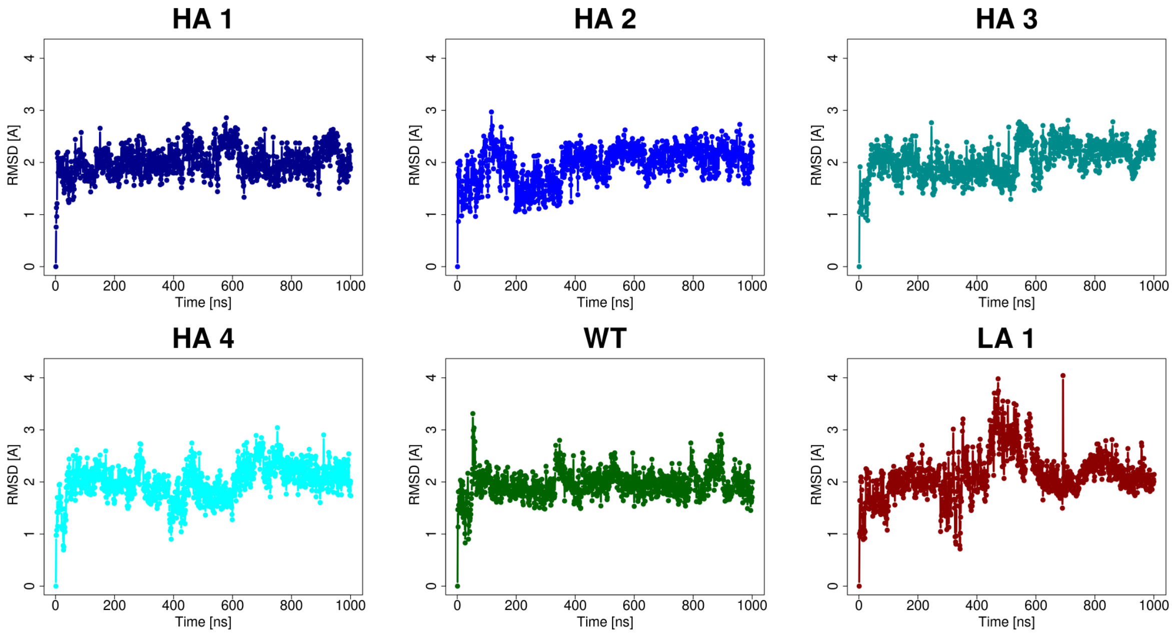

Figure S1: RMSD of the peptide backbone in the simulations of the Spike-peptide complexes

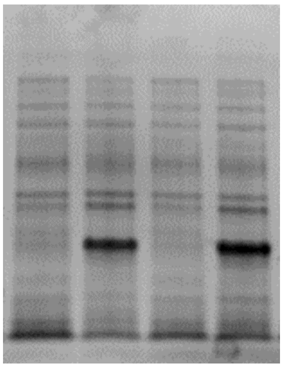

Figure S2: WT-HFt and HA1-HFt expression. Lane 1: WT-HFt pre-induction; lane 2: WT-HFt post-induction; lane 3: HA1-HFt pre-induction; lane 4: HA1-HFt post-induction.

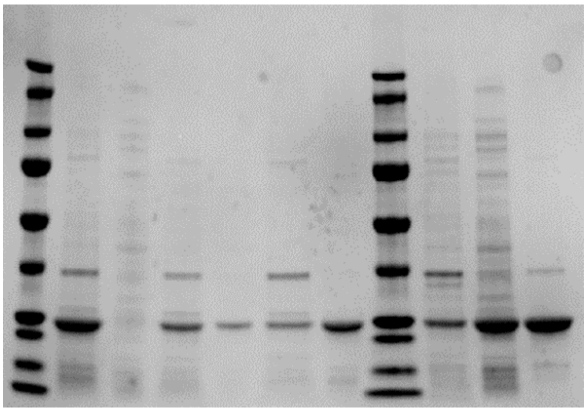

Figure S3: HA1-HFt and WT-HFt purification. Lane 1: Marker; lane 2: HA1-HFt Sonication insoluble fraction; lane 3: HA1-HFt sonication soluble fraction; lane 4: HA1-HFt Insoluble fraction 0.5 M urea; lane 5: HA1-HFt soluble fraction 0.5 M urea; lane 6: HA1-HFt insoluble fraction 4 M urea; lane 7: HA1-HFt soluble fraction 4 M urea; lane 8: Marker; lane 9: WT-HFt sonication insoluble fraction; lane 10: WT-HFt sonication soluble fraction; lane11: WT-HFt 20% ammonium sulfate.
